# Development of in-vitro maturation protocol for rat oocytes; under simple culture vs co-culture with cumulus cell monolayer and its developmental potential via Parthenogenetic/artificial activation

**DOI:** 10.1186/s12917-020-02714-8

**Published:** 2021-01-22

**Authors:** Muhammad Joan Ailia, Yun-Kyong Jin, Hee-Kyoung Kim, Goo Jang

**Affiliations:** 1 Laboratory of Theriogenology, Department of Veterinary Clinical Science, College of Veterinary Medicine, Seoul National University, Kwanak-ro 1, Daehak-Dong, Kwanak-Gu, Seoul, 08826 Republic of Korea; 2 LARTbio corporation, Seoul, Republic of Korea 06226

**Keywords:** SD rats, Ovaries, Superovulation, Oocytes, In vitro maturation, Parthenogenesis

## Abstract

**Background:**

Murine is the most abundantly used as laboratory animal models. There has been a tremendous amount of research including; their evolution, growth, physiology, disease modeling as well as genomic mapping. Rats and mice are the most widely used among them. Although both rats and mice fall under the same category still both are different a lot too. As regarding in vitro maturation and development mouse studies are well established as compared to rats which still lies in the early phase of development. So, we tried to figure out rat oocytes in vitro maturation and their developmental potential by performing 3 experiments i.e. superovulation, in vitro Maturation as simple culture (COC’s only), and COC’s & cumulus cells co-culture, which later further developed using parthenogenetic activation after IVM. Female Sprague Dawley rat 3–4 week used for these studies, we hyper-stimulated their ovaries using PMSG and hCG 150 IU/kg each. After that, we collected ovaries via dissection and retrieved oocytes. We matured them in TCM 199 supplemented with FSH, Estrogen, EGF, and Pyruvate. After maturation, we activated them using two types of activators i.e. Ethanol 7%, Ionomycin. After that, we saw and compared their developmental potential in vitro.

**Results:**

Oocytes matured in COC’s and Cumulus cell monolayer co-culture (59% ± 4*) showed significantly more even growth and extrusion of the first polar body as compared to the COC’s only culture (53.8 ± 7%*). While oocytes activated using Ionomycin showed more promising development until 8 cells/blastocyst level compared to ethanol 7%.

**Conclusion:**

we concluded that COC’s and cumulus monolayer co-culture is better than COC’s only culture. Cumulus monolayer provides extra aid in the absorption of nutrients and supplements thus providing a better environment for oocytes growth. Also, we concluded that matured oocytes showed more developmental capacity after activation via ionomycin compared to ethanol.

## Background

Murine are widely used laboratory animal because of their easy to handle size and fast growth rate. Rat is preferable because of its ideal size as compared to the mouse as well as its physiological more resemblance with humans thus used for many biomedical and genetic research [1]. Rat models have an edge because of its accurate representation of human diseases thus, widely used in studies like hypertension [2], atherosclerosis [3], and Huntington disease [4], etc. Not only rat provides accurate pathological data but also rat has widely diverse Genomic data [5] which provides a promising area that still needs to be explored, understands, and research. All this provides rat disease models edge over other lab animal models.

With the advancement in genome engineering and diverse genomic data [5] of rat still pending to be explored the creation of humanized rats is one of the possible field areas yet to be studied completely. Since the development of mouse embryonic stem cells (ESC) [6, 7] we have seen a rapid advancement in the production of genetically modified mouse models [8, 9]. Thus, we see a diverse and vast variety of mouse models available for research to date.

But as we discussed earlier rat models have an edge over mouse models because of comparatively easy handling and more accurate physiological and pathological data representation. So, as a vacuum for transgenic rat production developed, the need to produce genetically engineered rats increased. Over the years many precise genome modification tools and methods been developed for example; Targeted endonucleases, including zinc-finger nucleases (ZFNs) [10], transcription activator-like effector nucleases (TALENs) [11], and the clustered regularly interspaced short palindromic repeat (CRISPR)/CRISPR-associated (Cas) system [12].

In vitro maturation may be the answer to our problems and provides us the opportunity to maximize the potential of transgenic animal production [13] but with ease comes hurdles as well. Mouse oocytes maturation protocol is well studied compared to rat counterparts. Thus, we can find research data dating back to the 1970s either in terms of in vitro maturation and meiotic progression [14], mouse oocytes development in various culture systems [15] and studies regarding oocytes development, fertilization, or embryo development [16], etc. There are many reasons for that i.e. either more popularity of mouse as a laboratory animal, ease of availability, and focus of study. But one of the reasons in vitro maturation is long ignored and researchers still rely on conventional in vivo practices in rats because the quality of IVM oocytes is lower than in vivo counterparts [17, 18]. Nuclear maturation is evident but cytoplasmic maturation doesn’t go that well [19].

To overcome these challenges many biologists tried to understand the physiology of COC’s maturation in rats and many important suggestions were advised over the past decade. Rat oocytes require special signaling from the cumulus cells, follicular somatic cells, and bidirectional communication is necessary for successful maturation [20, 21]. Thus, many scientists tried to mimic in vivo conditions and advised co-culture for the proper maturation of oocytes [22].

One of the most popular co-culture is the development of oocyte in cumulus clumps [23, 24]. Cumulus and serum played an essential role in normal cytoplasmic maturation and subsequent developmental capability of rat oocytes [25]. In these studies, we tried to further evaluate and explore in vitro maturation of rat oocyte under co-culture, tried to assess their development using parthenogenetic activation using two activation chemicals Ionomycin and 7% ethanol.

## Methods

All reagents were purchased from Sigma-Aldrich Co. LLC. (Missouri, USA) unless otherwise specified.

### Care and use of animals

SD rats used in this study were purchased by Orient-bio (Republic of Korea). All-female rats aged 3–4 weeks were maintained in 24 ± 2 °C, 50% humidity, and 12:12 h light-dark cycle, a sample size of 6.5 ± 2 are maintained. All animal care and experiments were approved by the Institutional Animal Care and Use Committee (No.160719a-2-7) and performed under the guideline of Seoul National University.

### Euthanasia

All sample rats were euthanatized humanely by anesthesia (alfaxanole 10 mg /kg) accompanied by cervical dislocation under guidelines and training of Seoul National University.

### COC collection

The rats were injected PMSG (150 IU/kg) and hCG (150 IU/kg) (HCG, Daesung microbiological labs, Gyenggi, Republic of Korea) Intra peritoneally 48 h and 16 h prior to dissection respectively. Rats were anesthetized using Alfaxan (Jurox inc.) 10 mg/kg body weight IM (Intramuscular). The surgical site is disinfected using Ethanol 70%, an incision was given on linea alba, ovaries were retrieved and dissected and stored in pre-warm PBS solution. Later collected ovaries were sliced using a scalpel blade in M2 media (Special rat oocytes media). The released oocytes were then collected in 4 well plates containing 500 ul M2 solution in each well. Only germinal vesicle oocytes which have 3 layers unexpended cumulus layers and balanced cytoplasm were collected. They were washed three times in M2 solution and then incubated in pre-prepared TCM 199 media for maturation.

### In vitro maturation of oocytes

**Fig. 1:**
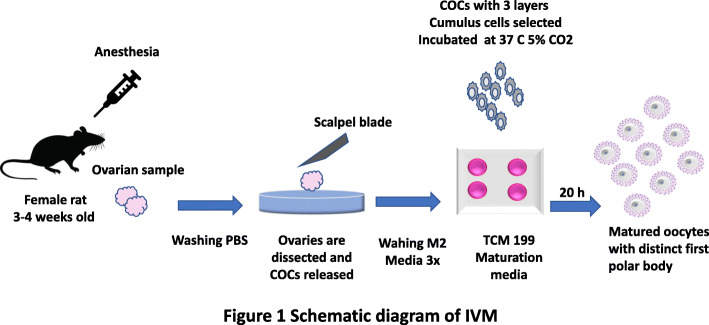
Schematic diagram of in vitro Maturation protocol, starting from superovulation via PMSG and hCG 150 IU 48 and 16-h prior sample collection respectively. Ovaries are collected via surgical incision and then dissected in M2 washing media, oocytes with 3 layers of intact cumulus cells are selected. They then incubated for 20 h in Co-culture or simple culture containing culture media TCM 199 supplemented with Fetal calf serum 10% volume by volume, estrogen 1 μg/ml, EGF 10 ng/ml, Cysteamine 100uM, Sodium pyruvate 0.45 mM, FSH 5 μg/ml. Figure is created by author himself using miscrsoft power point software and own all rights to it

Oocytes were matured in TCM 199 (cat. M5017) which was supplemented with; Fetal calf serum 10% volume by volume, estrogen 1 μg/ml, EGF 10 ng/ml, Cysteamine 100uM, Sodium pyruvate 0.45 mM, FSH 5 μg/ml. 4 well plate is used as maturation container. In each well 500 ul of TCM 199 plus supplementation, media is used. Oocytes collected from ovaries were first washed 3 times in M2 media than one time in TCM 199 media. After that, they are incubated in above mentioned supplemented TCM 199 media for 20 h at 37 °C temp. The quantity of 25–40 oocytes per/well is maintained (Fig. 1).

### Cumulus mono cell layer production

Cumulus cell monolayer is developed to co-culture it with oocytes to mimic the in vivo physiological conditions. For Cumulus cell monolayer development cumulus cells after denuding of oocytes are recovered in 1 ml Eppendorf tube. Then it’s centrifuged for 100 s at 370 g, the supernatant solution was aspirated and replaced with PBS. The procedure is repeated 3 times to wash cumulus cells. Pre-warmed DMEM supplemented with 10% volume by volume Fetal calf serum is used as culture media. Cells were grown until the completion of the monolayer; the media was replaced every few days. Later this monolayer is used to co-culture freshly obtained oocytes from the ovaries.

### Oocyte activation and culture

After 20 h of incubation oocytes, growth and maturation were checked. The Cumulus cell growth and extrusion of the polar body are preliminary morphological quality determinants. The TCM 199 media is aspirated using an aspirator and 0.1% hyaluronidase 500 ul is poured into each well to denude the oocytes. The plate was incubated for 3 min for successful distribution and effective denuding. The pipette is set to 50 ul and slow pipetting is done to detached cumulus cells from oocytes. After denuding oocytes were transferred to a fresh plate containing 500 ul of M2 media, then after washing twice the oocytes were then activated.

### Parthenogenetic activation

For activation 2 different type of activation chemicals are used;

#### Ethanol 7%

Seventy ul 100% ethanol is mixed in 930 ul of M2 to make 7% Ethanol solution. Oocytes were incubated in freshly made 7% ethanol solution for 3 min and then washed in M2 solution. After that incubated in DMAP 2 mM for 4 h.

#### Ionomycin

Denuded oocytes were activated in ionomycin solution for 4 min. Ionomycin is sensitive to light thus the procedure was done in a dark room with minimum microscopic light. Later to avoid light either wrap it with aluminum foil or incubate in the incubator for 4 min. Then wash activated oocytes in M2 and incubate in DMAP 2 mM for 4 h.

### In vitro embryo culture

**Fig. 2:**
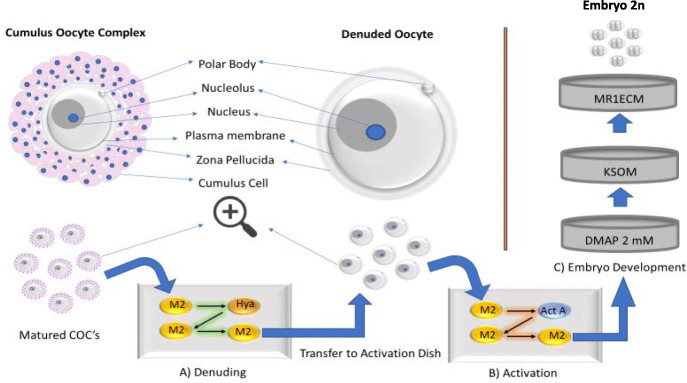
Schematic diagram of parthenogenetic activation and procedure until embryo development (2 cells). The main phases involved denuding, activation using either Ionomycin or ethanol 7%, and development in growth media till 2 cell embryos. Figure is created by author himself using Microsoft power point software and owns all rights to it

Incubated oocytes were then washed again using M2 media and transferred to 200 ul drop of KSOM media covered with an oil film, where they incubated for a further 20 h. After 20 h activated oocytes were observed and those with the second polar body or 2 cells were transferred to 100 um MR1ECM media (ARK Resource, Kumamoto, Japan). Twenty-five activated oocytes were kept per drop. Observed for further development, within 2–3 days oocytes usually reach 8 cells and blastocyst stage respectively. The procedure can be seen in the illustrated schematic diagram (Fig. 2).

### Statistical analysis

Data were analyzed by independent t-test (IBM SPSS 23). Results are mentioned as the mean ± standard deviation. A probability of *P* < 0.05 was considered to be significant.

## Results

### Oocyte maturation

**Fig. 3:**
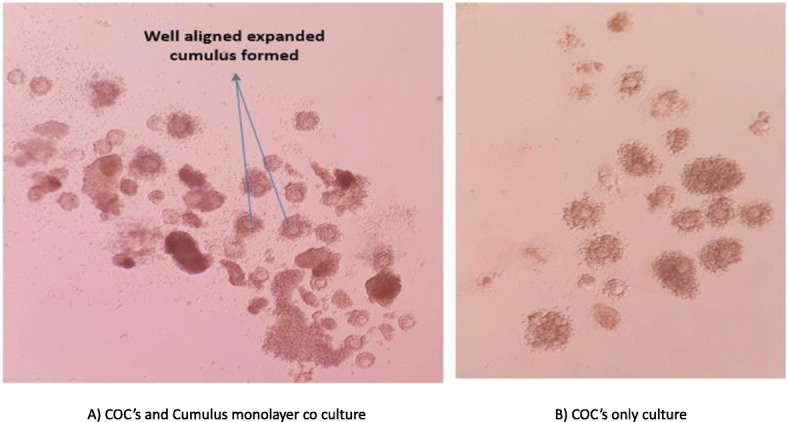
Comparison between Oocytes matured in the presence of Cumulus mono cell layer (**a**) vs Oocytes matured in simple culture (**b**).

Oocyte maturation was checked 20 h. post-incubation in TCM 199 media plus supplementation. The even growth of cumulus cell layers and even cytoplasm, as well as extrusion of the first polar body, were the initial markers of the development. Oocytes matured in cumulus cell monolayer co-culture showed more even growth and extrusion of the first polar body as compared to the normal culture. (Fig. 3).

### Parthenogenetic activation

**Fig. 4:**
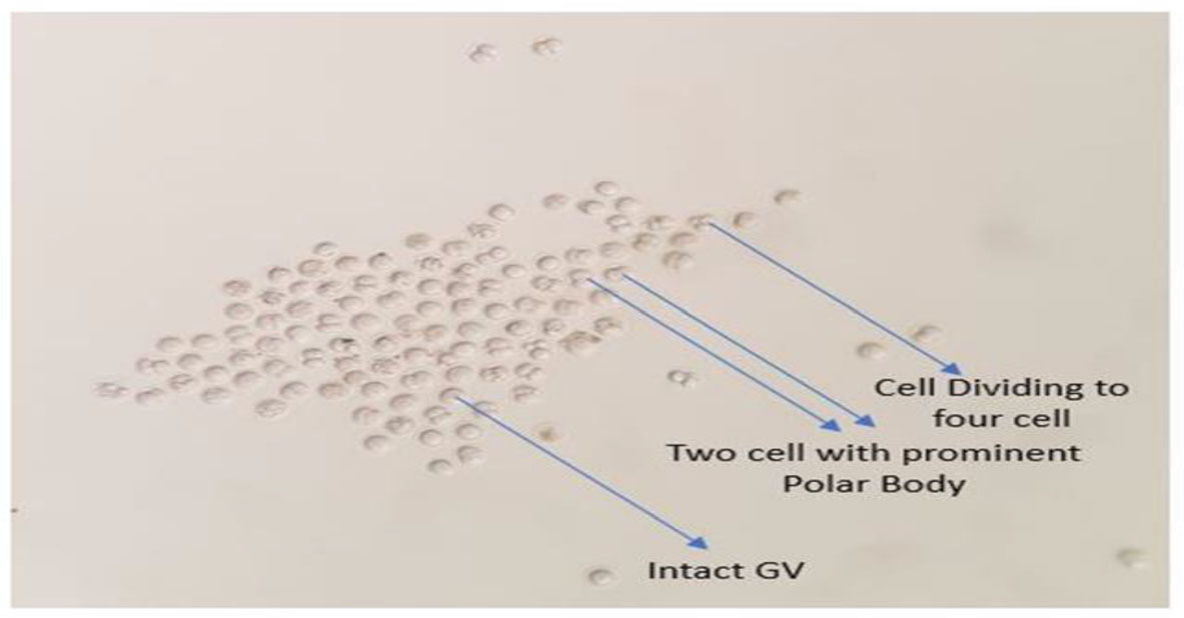
Activated oocytes post 20-h incubation, we can see dividing oocytes, 2 cells, and 4 cells

**Fig. 5:**
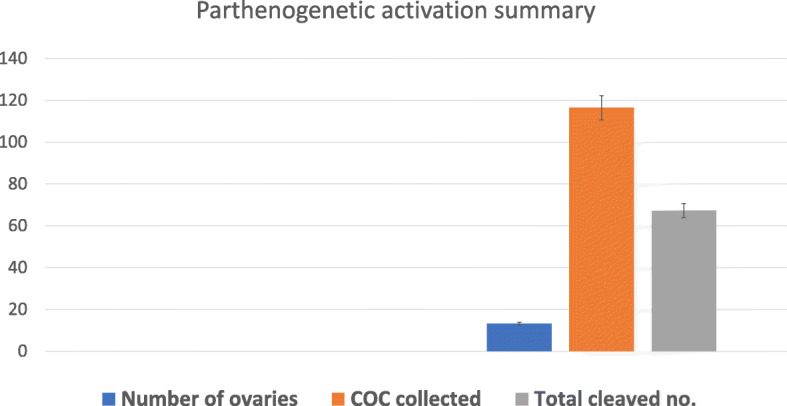
Parthenogenetic activation summary a total of an average of 116.42 ± 66.13 oocytes were activated using both methods i.e. Ethanol 7% and Ionomycin. 57.0% ± 0.05% oocytes Shown active cleavage after 20 h of incubation

**Fig. 6:**
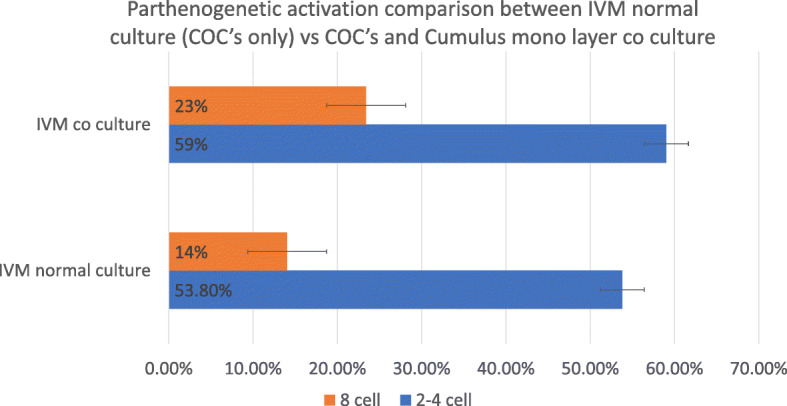
Comparison between in vitro maturation under simple and co-culture conditions. Showing significantly better development in co-culture compared to simple culture

The initial development of embryos was almost similar. Almost both methods of parthenogenetic activation have shown two cell division 20 h. post-activation (Fig. 4). Ethanol 7% shown fragmentation after 8 cell stages while Ionomycin has shown proper development till BL formation. On average 116.42 ± 66.13 oocytes were activated using both methods 57% ± 0.05% Shown active cleavage after 20 h of incubation as can be seen in Fig. 5). We see a significant improvement of development post-activation in co-culture with 59% ± 4 two cell development while 23% ± 2 at the eight-cell stage as can be seen in Table 1 and Fig. 6.

**Table 1:** Comparison between IVM methods Simple vs Co-culture

Donor	Method of IVM	The average number of Oocytes¹	Number of 2-4 cell (%) ¹	Number of 8 cells (%) ¹
SD Female Rat 3-4 weeks old	Simple culture	65.0 ± 1	53.8 ± 7^a^	14.0 ± 7^a^
	Co Culture	52.0 ± 2	59.0 ± 4^b^	23.0 ± 2^b^

¹Values of results were expressed as the means ± SD

^a-b^Values in the same column with different superscripts are significantly different (*P* < 0.05)

## Discussion

In this study, we tried to evaluate and develop basic guidelines for in vitro maturation protocol in rat oocytes. We tried to evaluate oocyte quality based on different markers i.e. expansion of the cumulus layer, the formation of polar bodies, cytoplasmic equilibrium (equal distribution of cytoplasm), and post parthenogenetic activation development. Our studies showed that those oocytes grown in co-culture with cumulus cell clumps showed better development than COC’s only culture. Many studies show that cytoplasmic maturation is crucial for the development of oocytes [26, 27]. One of the most important markers of cytoplasmic maturation is meiotic progression, redox state, and post-fertilization or activation development [28]. Previous studies have shown the metabolic and protective role of cumulus cells, for example, cumulus cells can reduce cystine to cysteine, enhance the uptake of cysteine cumulus cells can reduce cystine to cysteine [29], and increase the intra-oocyte glutathione level and protect the oocytes against oxidative stress [30]. Cumulus cells can also metabolize glucose to pyruvate that can be used by the oocyte to improve cytoplasmic maturation [31]. There are many ways of parthenogenetic activation and can be induced both physically as well as chemically. The key is to mimic the physiology involved during the fertilization process i.e. Conveying Ca2+ signals to the metaphase-II arrested oocytes. For that purpose, many different modes of stimuli are being studied for example, Electric stimuli are long used to induce exogenous Ca2+ into the cytoplasm of mature oocytes [32]. Also, many chemical activation agents have been studied as well like calcium ionophore, ethanol, and ionomycin they all increase intracellular Ca2+, which leads to activation of mammalian oocytes [26, 33]. Parthenogenetic activation opens a whole world of embryo development, either its SCNT, ISCI, or development of maternal genes only embryonic stem cells. It’s a field yet to be explored and exploited. Specifically, concerning rat parthenogenetic activation and embryo development is least understood as well as developed. Thus, based on previous studies we tried to develop an IVM co-culture protocol that may provide a key for the future development of transgenic rats in the in vitro environment and tried to access their developmental potential based on parthenogenetic activation, our studies can act as basic model to further study rat oocytes IVM and their developmental potential.

## Conclusion

Our studies have shown that a co-culture is the best protocol for IVM and ionomycin shows promising results in the activation of IVM oocytes. As demand for transgenic rats is increasing rapidly in vitro maturation in rats can answer to the humane and ethical production of transgenic rat models. Also, Parthenogenetic Activation of in vitro matured oocytes can help us develop ESC ethically as it doesn’t involve the manipulation of a fertilized embryo. In short, IVM is a broad useful area that still needs to be studied, explored, utilized, and conquered.

## Data Availability

The datasets used and/or analyzed during the current study available from the corresponding author on reasonable request.

## Abbreviations

ART: Assisted reproduction technology
COC: Cumulus-oocyte complex
DMSO: Dimethyl sulfoxide
DNA: Deoxyribonucleic acid
ECG: Equine chorionic gonadotropin
ESC: Embryonic stem cell
FSH: Follicle-stimulating hormone
h: Hours
hCG: Human chorionic gonadotropin
IP: Intraperitoneal injection
IV: Intravenous injection
N.B: Nota bene (Note well)
RNA: Ribonucleic acid
SCNT: Somatic cell nuclear transfer
SD: Sprague Dawley
